# Lightweight Concrete Produced Using a Two-Stage Casting Process

**DOI:** 10.3390/ma8041384

**Published:** 2015-03-25

**Authors:** Jin Young Yoon, Jae Hong Kim, Yoon Yi Hwang, Dong Kyu Shin

**Affiliations:** 1 School of Urban and Environmental Engineering, Ulsan National Institute of Science and Technology, 50 UNIST-gil, Ulju-gun, Ulsan 689-798, Korea; E-Mail: dc4408@naver.com; 2 Structure Research Department, Corporate Technology Institute, Hyundai Heavy Industries Co., Ltd., Ulsan 682-792, Korea; E-Mails: hyn1014@hhi.co.kr (Y.Y.H.); dongkyu.shin@hhi.co.kr (D.K.S.)

**Keywords:** lightweight concrete, two-stage concrete, volume fraction, aggregate, grout

## Abstract

The type of lightweight aggregate and its volume fraction in a mix determine the density of lightweight concrete. Minimizing the density obviously requires a higher volume fraction, but this usually causes aggregates segregation in a conventional mixing process. This paper proposes a two-stage casting process to produce a lightweight concrete. This process involves placing lightweight aggregates in a frame and then filling in the remaining interstitial voids with cementitious grout. The casting process results in the lowest density of lightweight concrete, which consequently has low compressive strength. The irregularly shaped aggregates compensate for the weak point in terms of strength while the round-shape aggregates provide a strength of 20 MPa. Therefore, the proposed casting process can be applied for manufacturing non-structural elements and structural composites requiring a very low density and a strength of at most 20 MPa.

## 1. Introduction

Structural lightweight concrete has been widely used following advances in production technology for lightweight aggregates. The advantage of lightweight concrete is reduced dead load for structures. Thus, the required cross-section of columns, amount of steel, and foundation load can decrease, given that lightweight concrete has acceptable performance in terms of strength [1,2,3,4]. Consequently, the application of lightweight concrete in long-span bridges and high-rise buildings can reduce building costs [3,5,6]. Typically available lightweight aggregates are usually produced using sintered fly ash, expanded clay and expanded shale [2,3,5,6,7]. They inherently have a large number of pores, which results in lower strength, stiffness and greater deformability [2,6]. Thus, weak lightweight aggregates affect the strength and failure of concrete [6]. However, Wasserman and Bentur [5] reported that higher strength aggregates do not necessarily lead to higher strength concrete, and Ke *et al.* [6] demonstrated that volume fraction of lightweight aggregates is in inverse proportion to compressive strength. Therefore, the strength of lightweight concrete can be controlled via the properties and volume fraction of lightweight aggregates as well as the water-to-cement ratio [1,5,6,8].

Various studies have investigated the physical and mechanical properties of lightweight concrete with the use of lightweight coarse aggregates and normal weight fine aggregates [6,7,8,9,10,11]. Unit weight of lightweight concrete is determined by the types and content of lightweight aggregates. Nguyen *et al.* [2] and Kockal and Ozturan [10] studied the strength and modulus of ordinary vibrated lightweight concrete. They reported that the density and aggregate volume fraction were 1,650 kg/m^3^ and 42%, respectively, for ordinary vibrated lightweight concrete. The volume fraction of lightweight aggregates in ordinary vibrated concrete was up to 45% [6,10]. However, that of self-compacting concrete (SCC) decreased 25% to 35% [7,11]. To get enough flow for self-consolidation, its aggregate contents are reduced to decrease viscosity. This method for mix proportioning is also valid for normal-weight SCC. Bogas reported that the dry density of ordinary vibrated lightweight concrete was reduced to as low as 1,750 kg/m^3^ according to the mix proportion, but lightweight SCC could not achieve a density below 1,950 kg/m^3^ with the use of given lightweight aggregates of Arlita [9]. The research that followed recommended a coarse aggregate volume fraction of 30% to 34% for lightweight SCC, a range that reportedly provides segregation resistance [12]. Kanadasan and Razak [11] and Guneyisi *et al.* [7] reported lightweight SCC having a density of 2,042 kg/m^3^, with a volume fraction for lightweight aggregates of 31%. To sum up, the foregoing examples indicate that conventional mixing methods limit the density of lightweight concrete to about 1,700 kg/m^3^. If we maximize the volume fraction of lightweight aggregates in a mix, it is possible to further reduce the density of lightweight concrete.

This paper proposes a two-stage casting process to increase the volume fraction of lightweight aggregate, which results lower density. Given that the same-density lightweight aggregate is used, the proposed process produces much lighter concrete than the conventional mixing procedure. The process to cast a structural member is (1) placing lightweight coarse aggregates in the mold and (2) injecting cement grout into the interstitial voids between the preplaced aggregates. The process involves producing preplaced concrete, but this has not been realized for lightweight concrete in the literature [13]. Four types of lightweight aggregates were tested to show the feasibility of the proposed method. The physical and mechanical properties of samples produced by the proposed method were investigated to evaluate their performance. The effects of characteristics of lightweight aggregates, such as shape, size distribution and packing density, are also discussed.

## 2. Sample Preparation

**Figure 1:**
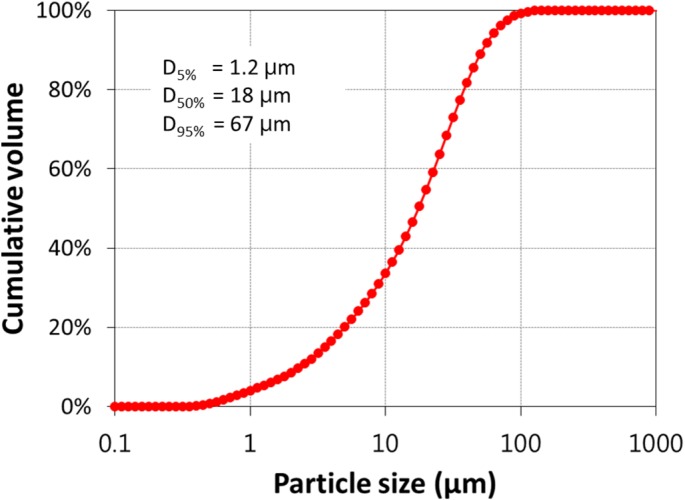
Particle size distribution of Portland cement.

Portland cement was used for making lightweight concrete. Its specific gravity was 3,150 kg/m^3^, and the Blaine specific surface area was 335 m^2^/kg. They were measured in accordance with ASTM C188 [14] and ASTM C204 [15], respectively. The oxide composition of the used cement is reported in Table 1. Its particle size distribution is given in Figure 1, measured using a laser diffraction technique. Diluting and agitating the cement powder in 1% acetone solution deflocculated its particles. The laser diffraction captured each particle size and constructed the distribution.

**Table 1:** Oxide composition of cement.

Oxide	CaO	SiO_2_	Al_2_O_3_	MgO	SO_3_	Fe_2_O_3_	K_2_O	TiO_2_	Na_2_O
Percent (%)	65.8	17.6	4.4	3.2	3.4	3.4	1.1	0.3	0.2

**Table 2:** Types of lightweight aggregates and their properties.

Label	B700	EPS	ST700	AS700
Raw material	Clay	Polystyrene	Slate	Shale
Maximum size, mm	12	11	19	15
Mean size, mm	10	11	10	10
Oven dry specific density, kg/m^3^	1178	34.9	1358	1272
SSD * specific density, kg/m^3^	1383	34.9	1436	1458
Dry bulk density, kg/m^3^	692	15.8	769	772
SSD * bulk density, kg/m^3^	770	15.8	811	879
Shape	Spherical	Spherical	Irregular	Irregular
Water absorption	17%	0%	5%	14%

* SSD means saturated-surface dry condition.

**Figure 2:**
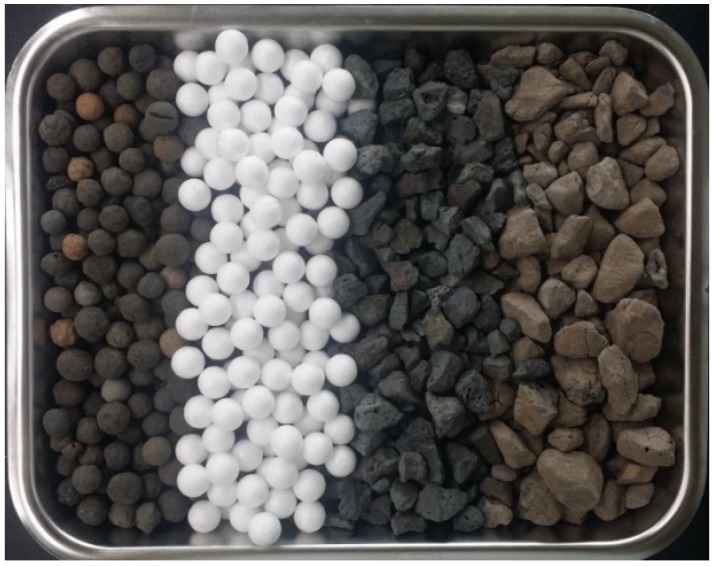
Shape of lightweight aggregates (B700, EPS, ST700, and AS700 from left to right).

**Figure 3:**
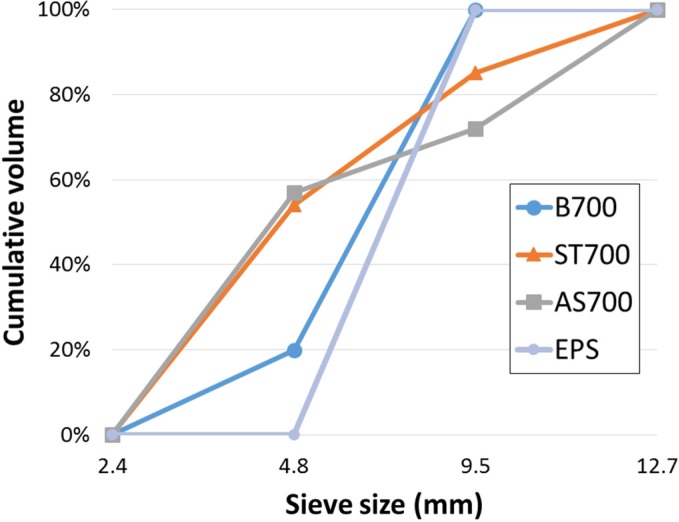
Gradation of lightweight aggregates.

A total of 4 lightweight aggregates were used. Their physical properties and detailed information are listed in Table 2, where they are labeled as B700, expanded polystyrene (EPS), ST700, and AS700. The mean size of all aggregates was similar, approximately 10 mm. Their shape and particle size distribution were different. B700 was expanded clay. Due to sintering blowup for its production, its shape was very spherical as shown in Figure 2 and roughly mono-sized as shown in Figure 3. Its specific density and loosely-packed bulk density in dry conditions were 1,178 kg/m^3^ and 692 kg/m^3^, respectively. The suffix label of 700 comes from its nominal bulk density of 700 kg/m^3^.

On the other hand, ST700 and AS700 had irregular shapes as shown in Figure 2. Their production first involved sintering expansion and then being crushed into a designated size, which also resulted in multi-size distribution up to the maximum 19 mm as shown in Figure 3. Their mean sizes were approximately the same as B700, 9.7 mm and 10.2 mm, respectively. Their specific densities in dry conditions were 1,358 kg/m^3^ and 1,272 kg/m^3^, respectively. Even though their nominal bulk density label was marked 700 kg/m^3^, their measurements in the lab were somewhat higher, 769 kg/m^3^ and 772 kg/m^3^, respectively. In summary, the two samples had adequately similar mean size and dry bulk density with B700, but their size distribution and shape were totally different.

EPS beads were commercially available. EPS is produced by heating expansive polystyrene. It is inherently composed of a closed-cell structure, and consequently it has very low density. The closed-cell structure also prevents water from being absorbed into the material. In this research, its specific density and bulk density were 34.9 kg/m^3^ and 15.8 kg/m^3^, respectively. The appearance of EPS was very spherical as shown in Figure 2, and its mean size was 11 mm. Therefore, it can be said that EPS has the same shape and dimension as B700, but is far lighter. Lightweight concrete incorporating EPS could have very low density, under 1,000 kg/m^3^.

**Figure 4:**
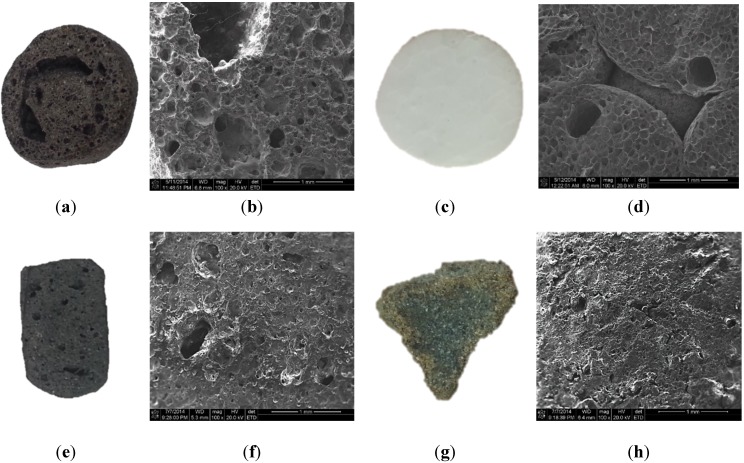
Cross section of lightweight aggregates and its microscopy image: (**a**,**b**) B700; (**c**,**d**) EPS; (**e**,**f**) ST700; (**g**,**h**) AS700.

The density and strength of lightweight aggregates are governed by their raw materials and production conditions. They have a sparse microstructure in common. A number of large pores exist inside, which results in lower density, lower strength and higher water absorption. To investigate inside lightweight aggregates, scanning electron microscopy (SEM) provided the microstructural images of aggregates cross section as shown in Figure 4a–h. There are many macro and micro voids in B700, with a rough texture. These pores reduce the weight of B700 and allow a large amount of water absorption. In the case of EPS, its inside is totally different from the others. There are no macro pores, but it was composed of circular honeycomb cells with micro voids. A high number of pores and its material properties make EPS lighter. The other lightweight aggregates, ST700 and AS700, also have smaller and fewer micro pores. Hence, both of them have comparatively higher specific density.

The mix proportion of each sample is described in Table 3. All samples were made in a cylinder with 100 mm diameter and 200 mm height. The curing temperature and relative humidity were maintained at 23 °C and 95%, respectively. Specifically, sample N1 is ordinary vibrated lightweight concrete showing a slump of 150 mm to 200 mm. Sample S6 is lightweight SCC with a slump flow of 625 mm. Both samples are control mixes produced by the conventional mixing procedure. In brief, that procedure was as follows: place cement and aggregates for dry mixing and add water to the mix. Stir unmixed materials for 1 min, and continue mixing for 2 min. Fine aggregate (normal weight) was the combination of washed sea sand and crushed sand. The fineness modulus, specific density, and water absorption of the fine aggregate were 2.58, 2,600 kg/m^3^ and 1.48%, respectively. The used coarse lightweight aggregates were saturated surface dried for mixing. The high-range water reducing admixture (HRWRA) was used for the SCC sample S6. It was polycarboxylate-based, and its solid content was 20%. In contrast, samples P2, P5, P6 and P7 were produced by applying the proposed two-stage casting process. The samples used oven-dried lightweight coarse aggregates and did not use any fine aggregates. Details on the casting process and the contents of the mix are described in the next section.

**Table 3:** Mix proportion for lightweight concrete. *w*/*cm*, water-to-cement ratio.

Label	*w*/*cm*	Water (kg/m^3^)	Cement (kg/m^3^)	Aggregate (kg/m^3^)		HRWRA (kg/m^3^)
				Coarse	Fine	
N1	0.42	277	660	416	416	–
S6	0.35	297	849	351	351	3.51
P2 (B700)	0.42	235	559	692	–	1.75
P5 (EPS)	0.42	246	586	19	–	1.84
P6 (ST700)	0.42	247	587	769	–	1.81
P7 (AS700)	0.42	223	532	772	–	1.72

## 3. The Proposed Two-Stage Casting Process

**Figure 5:**
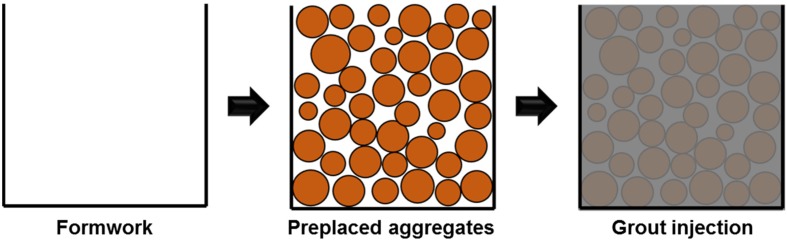
Illustration of the two-stage casting process.

Maximizing the volume fraction of lightweight aggregates allows us to have the lowest density of lightweight concrete. The two-stage casting process is proposed for this purpose: (1) placing the lightweight aggregates in an empty mold to produce full packing and (2) filling the interstitial void spaces in the mold with cement grout. Figure 5 illustrates the proposed casting process.

The details of the mixing sequence for the samples in Table 3 are as follows: A mold is filled with the dried lightweight aggregates. A cement grout (paste) prepared by 5 min shearing is poured into the mold until it completely fills the void left between aggregates. The water and cement contents in Table 3 corresponds to those for the cement grout to produce 1 m^3^ lightweight concrete. Therefore, the water-to-cement ratio (*w*/*cm*) in the table is the ratio of cement grout. Assuming that the oven-dried lightweight aggregates, for example B700, in the mix absorb as much water as possible, the 17% water content decreases considering its water absorption as reported in Table 2. The effective *w/cm* of the sample P2 would be 0.21 rather than the initial 0.42, consequently. Therefore it should be noted that Table 3 reports the total contents for weighing constituent materials based on the oven-dried aggregate condition. It does not indicate the effective *w/cm* of binding materials. The grout used does not have fine aggregates in this study. Because the internal gaps between lightweight aggregates were narrow, the use of fine aggregates in grout may limit its filling. In future studies, the use of fine fillers such as silica powder could be considered if necessary.

## 4. Experimental Results and Discussion

### 4.1. Volume Fraction of Lightweight Aggregates

**Figure 6:**
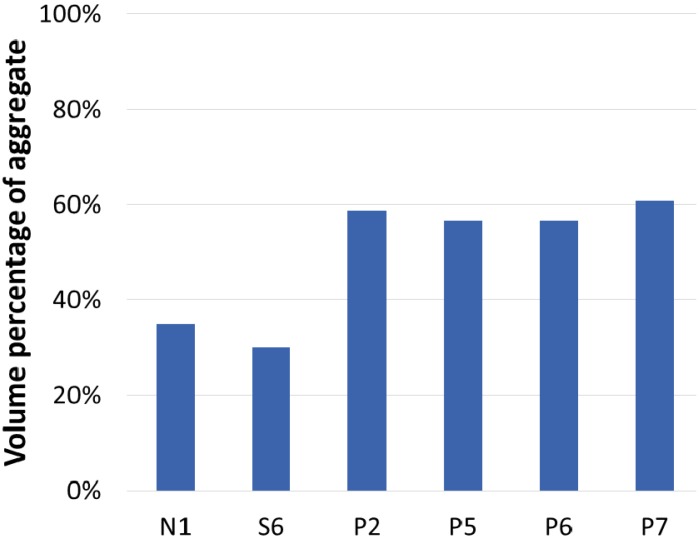
The volume fraction of lightweight aggregates in each sample.

The volume fractions of ingredients of the samples are given in Figure 6. The ordinary vibrated concrete N1 and lightweight SCC S6 incorporated the same fine and coarse aggregates of B700 sample. As shown in Figure 4, lightweight aggregates had a number of pores inside, which reduced its density. Therefore, the volume fraction of lightweight aggregates in concrete determines the unit weight of hardened concrete. High-fluidity mix S6 had a lower content of lightweight aggregates than ordinary mix N1: 30% *vs.* 35%. The fluidity and self-compacting ability of a mix were obtained by the use of fine materials and incorporating chemical admixture, such as HRWR admixture [16]. Therefore, it increased the cement content and reduced the volume fraction of coarse aggregates. ACI 237 committee on SCC reports coarse aggregates generally use 28% to 32% by volume of the mix, which is the pertinent range for producing SCC [17]. Mix S6 (30%) in the study followed the recommended range, and the ordinary concrete mix N1 (35%) comparatively had more aggregates. As a result, a mix design for producing lightweight SCC had a lower volume fraction of lightweight aggregates and thus higher unit weight. Note that the hardened density of ordinarily concrete N1 and SCC S6 were 1,842 kg/m^3^ and 1,933 kg/m^3^, respectively.

The above results confirm that lightweight concrete incorporating a higher amount of lightweight aggregates has lower unit weight. Thus, in order to reduce the unit weight of concrete, it is necessary to increase the volume fraction of lightweight aggregates in the mix. Mixes P2, P5, P6, and P7 in the study were produced using the proposed two-stage casting process, in which the volume fraction of lightweight aggregates was maximized. The mix design of lightweight preplaced concrete samples of P2, P5, P6, and P7 is reported in Table 3, where the lightweight aggregates used are listed in parenthesis for each label.

Sample P2 used lightweight aggregate of B700. Packing density of B700 in a 100 mm-diameter by 200 mm-height cylinder mold was 59%, and consequently its volume fraction in P2 was the same, 59%. The preplaced casting method produced a higher volume fraction of B700 in P2 than samples N1 and S6 which also used the same aggregates. The hardened density of P2 was 1,507 kg/m^3^, much less than that of N1 or S6. For producing much lighter concrete, another aggregate (EPS) was used. The use of EPS in P5 produced the lowest density of lightweight concrete, 755 kg/m^3^.

The samples P2 (B700) and P5 (EPS) have similar volume fractions of 59% and 57%, respectively. Slight increase with B700, about 2%, presumably comes from a slight size difference in aggregate B700. Figure 3 shows 20% of B700 is smaller than 9.53 mm, but EPS has perfect mono-size as shown in Figure 2 and Figure 3.

Samples P6 and P7 were produced using irregularly shaped aggregates ST700 and AS700, respectively. Their sizes are graded as shown in Figure 3. The packing density of graded particles was reportedly higher than that of mono-size particles [18]. However, in the study, ST700 showed the same packing density as EPS, 57%. Its irregular shape is thought to prevent a decrease in void space in the mold. Nevertheless, its graded size decreases the void size between aggregate particles smaller than B700. The packing density of AS700 was 61%, which supports the idea that graded aggregate size increases the packing and decreases the void space. The hardened densities of P6 and P7 were 1,612 kg/m^3^ and 1,564 kg/m^3^, respectively. Their densities were higher than that of P2 (1,507 kg/m^3^). Even though the nominal bulk density of all lightweight aggregates are the same, 700 kg/m^3^ as indicated by their label, the dry specific densities of aggregates ST700 and AS700 are higher than B700. The higher specific density results in the higher density of samples P6 (ST700) and P7 (AS700).

### 4.2. Compressive Strength and Failure Mode

**Figure 7:**
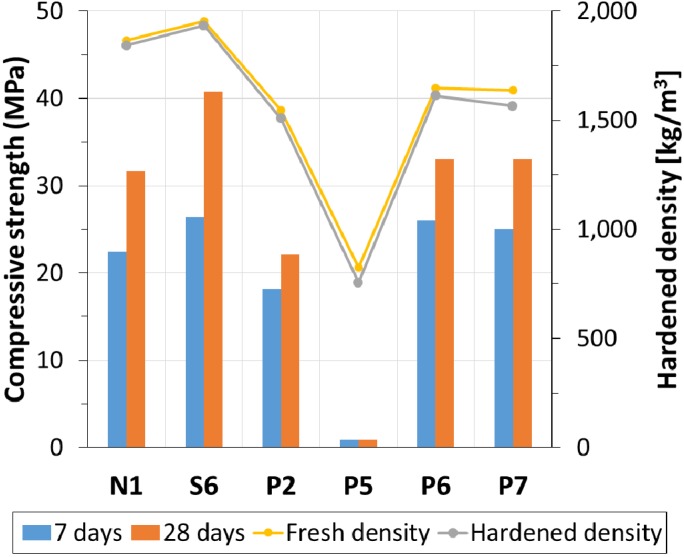
Compressive strength and density of lightweight concrete.

Figure 7 shows the compressive strength and density of the concrete samples. A proportional trend between density and strength of materials is clearly shown in the figure. A low-density concrete had low strength. Other studies also showed the proportional relationship between the compressive strength/elastic modulus and the unit weight of concrete [1,2]. All samples using the same lightweight aggregates, N1, S6, and P2, follow the proportional trend. For comparison, the compressive strength of each type is listed as follows: lightweight vibrated concrete N1 had a strength of 31.6 MPa at 28 days, lightweight SCC had 40.8 MPa, and lightweight preplaced concrete P2 had 22.1 MPa. However, the samples using different lightweight aggregates, ST700 and AS700, did not fall into the trend. Their strengths were higher than that of P2—33 MPa at 28 days. The strength improvement of P6 and P7 is caused by the quality of the aggregates used. In addition, the interlocking aggregates of P6 and P7 could also contribute to their strength increase. Both aggregates have irregular shape and multi-size distribution.

**Figure 8:**
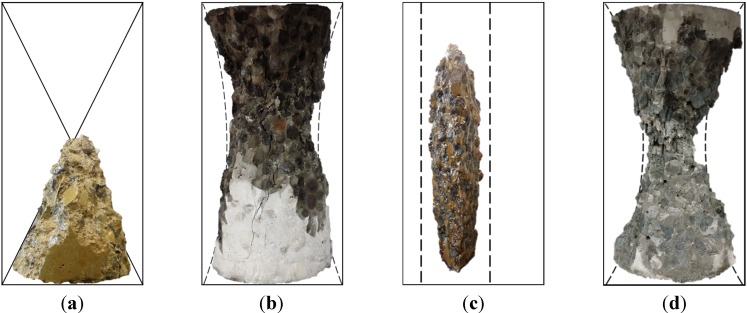
Failure mode: (**a**) normal concrete; (**b**) one with P2; (**c**) the other with P2; and (**d**) P6.

As shown in Figure 8, investigations on the failure mode of lightweight concrete were conducted. For comparison of the failure mode, the failure shape of normal-weight concrete under compression is shown in Figure 8a. This was produced using normal-weight aggregates. Its *w/cm* was 0.53 and the sand-to-total aggregates ratio was 0.49. The nominal density and 28-days compressive strength were 2,300 kg/m^3^ and 24 MPa, respectively. Generally, failure of normal concrete starts from the interfacial transition zone (ITZ), and no failure in aggregates were found on the fracture surface, as shown in Figure 8a. The failure under compression displays a steep diagonal cone shape due to shear failure of materials as expected. The shear failure is composed of initial ITZ cracking and its propagation on paste.

On the other hand, the types and amounts of lightweight aggregates determine the mechanical behavior of hardened concrete due to their low failure resistance. On the fracture surface of lightweight concrete, in contrast, many lightweight aggregates were destroyed as shown in Figure 8b–d. This influenced the failure mode of the concrete samples [6]. Figure 8b,c is fractured specimens of P2. They exhibit a less steep slope in cone, diagonal and even column-shaped failure. Half-split aggregates on the fracture surface indicate that the failure of the concrete sample starts from the inner space of lightweight aggregates (B700). Lower resistance in transverse strain due to the lightweight aggregates causes bulging of the cylinder sample [18]. When compression is applied to a sample, the aggregates consequently experience splitting tensile failure. A high volume fraction of lightweight aggregates makes the splitting tensile failure of aggregates being connected, which results in the column-shaped failure of a sample. When a small amount of lightweight aggregates was used for the SCC sample, S6, column-shaped failure was not observed.

In the case of a bit heavier aggregates ST700 and AS700, much smaller amounts were destroyed on the fracture surface in Figure 8d. Lightweight concrete (P2) made with those aggregates exhibits steep diagonal cone-shaped failure indicating more or less shear failure, like normal concrete. Ther are two possible reasons for the different shape of the failure: (1) higher strength of ST700 and AS700, because their specific density is higher than that of B700, providing higher resistance against splitting tensile failure [19]. A high strength aggregates lowers the Poisson’s effect and the bulging of the cylinder sample decreases. Transferring compression to lateral strain is consequently limited, which results in less tensile failure in the lateral direction; (2) irregular-form and multi-size particles of irregular-form aggregates with a high expected degree of aggregate interlocking provide higher strength for P6 and P7, which prevents the connection from experiencing splitting tensile failure for each grain. The phenomenon was found by Wasserman and Bentur [5], in which the strength of lightweight concrete was not always proportional to the strength of aggregates used. In addition, other experimental results showed that multi-size particles enhance stress distribution effectively through contact part of concrete composition, and increase its compressive strength [13]. When the volume of lightweight aggregates is maximized in the current samples, the compressive stress distribution through the cement paste is no longer effective. However, the transverse bulging due to the Poisson’s effect is effectively confined by the paste and enhanced interlocking of aggregates.

The effect of water absorption on strength needs to be discussed. The high water absorption of lightweight aggregates reportedly increases compressive strength because their saturation prior to mixing provides continuous water supply for internal curing [20]. In addition, the use of dried aggregates like in this study also contributes to increased strength considering the effective *w/cm*, lower than the initial mix proportion, and by eliminating internal bleeding on the ITZ [21]. As previously described, the mix proportion in Table 3 was not the effective *w/cm*. The effective *w/cm* for P2 (B700 having 17% water absorption) was 0.21 lower than the initial 0.42 from the previous calculation. The strength of sample P2 was lower than those of samples P6 (ST700 having 5% water absorption) and P7 (AS700 having 14% water absorption). A higher water absorption case (a lower effective *w/cm*, P2) produced the lower strength. Therefore, the effect of water absorption on the strength is marginal and the characteristics of aggregates are dominant in affecting the performance of lightweight concrete in the samples in the study.

Table 4 reports the dynamic moduli of P2, P6 and P7. The dynamic modulus of elasticity was measured according to ASTM C 215 [22]. A total 3 of 100 mm-diameter 200 mm-height cylinders were tested to determine the frequencies of longitudinal and torsional resonance. Lightweight concrete reportedly shows elastic modulus and dynamic modulus of elasticity are proportional to the density of concrete, and certainly a higher strength was obtained with a higher elastic modulus [23,24]. The trend has continued in the results of this study. However, it should be noted that the dynamic modulus of sample P6 is higher than that of sample P7 even though they produced the same strength of 33 MPa. Assuming that the dynamic modulus of each sample is proportional to the physical properties of the lightweight aggregates inside, the aggregate AS700 (for P7) is weaker than the aggregate ST700 (for P6). The high absorption and aggregate interlocking of AS700 in P7 compensate for the lower resistance in itself.

**Table 4:** Dynamic modulus of lightweight concrete at 28 days.

Type	Resonant Frequencies, kHz		Dynamic Modulus, GPa	
	Longitudinal	Torsional	Young’s	Shear
P2	7.20	4.59	12.0	4.9
P6	8.83	5.63	18.9	7.7
P7	7.86	5.08	15.2	6.4

### 4.3. Discussion on the Casting Process

**Figure 9:**
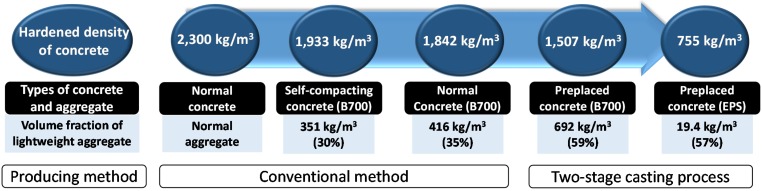
Flow chart of research for producing lightweight concrete.

Figure 9 shows a diagram of mix design methodology. Given that the same lightweight aggregate is used, density of lightweight concrete is determined by the volume fraction of lightweight aggregate. The mix design and casting process should be changed according to the desired volume fraction of aggregates. The volume fraction is minimized for producing SCC. The use of HRWR agent provides low viscosity for fluidity, but suspending aggregates in paste becomes difficult. Therefore, the volume fraction of lightweight aggregates is limited due to the stability problem. A sample of lightweight SCC has a density of 1,933 kg/m^3^ in the current study. Field application of lightweight SCC was recently discussed at an academic conference, the ACI 2014 Fall Convention [25,26,27,28,29]. Researchers reported lightweight SCC at approximately 2,000 kg/m^3^ is currently producible. Normally vibrated concrete has a higher yield stress and makes it possible to increase the amount of aggregate content. The volume fraction is increased by approximately 5% for a thick mix. Considering the type of lightweight aggregates, possibly supplied in the field, allows us to have more or less 1,800 kg/m^3^ [9]. A greater increase in the volume fraction was achieved by the proposed two-stage process. The volume fraction increased up to 59%. The density of lightweight concrete is minimized at 1,507 kg/m^3^ when the bulk density of lightweight aggregates is nominally 700 kg/m^3^. If we use lighter aggregates, a lower density of lightweight concrete is obviously expected. The lightest concrete by far (P5) was such an example even though it did not gain meaningful strength development for 28 days. Its final strength was 0.9 MPa, which is not acceptable for structural concrete. Nevertheless, it can be considered for floating structures and insulation materials with a very low specific density of 755 kg/m^3^ [30].

The samples produced by the two-stage casting process can be classified under EN 206-1 [31]. The strength of a 100 mm-diameter cylinder is 4% higher than that of a standard cylinder, so multiplying the correction factor of 0.96 produces the strength of the concrete samples used, shown in Figure 7. Ordinary vibrated lightweight concrete N1 is LC30/D2.0; lightweight SCC S6 is LC40/D2.0; lightweight two-stage concrete P2 is LC20/D1.6; and others made with different lightweight aggregates P6 and P7 are LC30/D1.8 and LC30/D1.6, respectively. Due to very low unit weight and compressive strength, P5 cannot be classified.

All concrete samples used the same *w/cm* of 0.42, but S6 used a different *w/cm* of 0.35, which is much lower than other concrete samples. Thus, the compressive strength of S6 is high, mainly due to a short volumetric distance for cement particles and a low volume fraction of aggregates. When aggregates are strong enough to endure compressive strength, good packing density improves stress distribution through aggregates. Hence, crack propagation starts along the ITZ [19].

## 5. Conclusions

The conventional method of producing lightweight concrete takes into account the workability and aggregate segregation of the fresh mix. Therefore, conventional mix design is limited by the retainable volume fraction of lightweight aggregates, and consequently the achievable lowest density of lightweight concrete is also limited. It is generally 2,000 kg/m^3^ for self-compacting mix and 1,800 kg/m^3^ for ordinary vibrated mix. The two-stage casting process maximizes the volume fraction of lightweight aggregates and consequently produces a lightweight concrete with the minimum density. A sample produced with aggregates that have a bulk density of 700 kg/m^3^ exhibited a density of 1,500 kg/m^3^ to 1,600 kg/m^3^ and a compressive strength of 20 MPa to 30 MPa nominally. Moreover, ultra-lightweight concrete at 750 kg/m^3^ can be produced.

The lightweight two-stage concrete is composed of fully packed lightweight aggregates, which changes the failure mode of the cylinder under compression. The weakest phase in the microstructure of lightweight concrete is not the ITZ, but the lightweight aggregates themselves. The splitting tensile failure of lightweight aggregates is connected in the vicinity and then the whole cylinder bursts under compression. The use of multi-sized or irregular-form lightweight aggregates increases the aggregate interlocking, and consequently the compressive strength returns its failure mode to a general diagonal cone shape.
